# Biological Activities of 2-Mercaptobenzothiazole Derivatives: A Review

**DOI:** 10.3797/scipharm.1204-27

**Published:** 2012-06-18

**Authors:** Mohammed Afzal Azam, Bhojraj Suresh

**Affiliations:** Department of Pharmaceutical Chemistry, J. S. S. College of Pharmacy, Ootacamund-643001, Tamil Nadu, India.

**Keywords:** 2-Mercaptobenzothiazoles (MBT), 2-Sulfanyl-1,3-benzothiazoles, Antimicrobial activity, Anti-inflammatory activity, Heat shock protein 90, Monoamine oxidase

## Abstract

2-Mercaptobenzothiazoles are an important class of bioactive and industrially important organic compounds. These compounds are reported for their antimicrobial and antifungal activities, and are subsequently highlighted as a potent mechanism-based inhibitor of several enzymes like acyl coenzyme A cholesterol acyltransferase, monoamine oxidase, heat shock protein 90, cathepsin D, and c-Jun N-terminal kinases. These derivatives are also known to possess antitubercular, anti-inflammatory, antitumor, amoebic, antiparkinsonian, anthelmintic, antihypertensive, antihyperlipidemic, antiulcer, chemoprotective, and selective CCR3 receptor antagonist activity. This present review article focuses on the pharmacological profile of 2-mercaptobenzothiazoles with their potential activities.

## Introduction

**Fig. 1.**
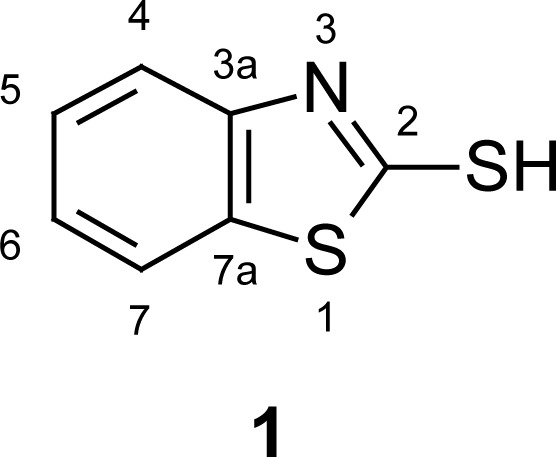
Structure of 2-mercaptobenzothiazole

The main objective of organic and medicinal chemistry is the design, synthesis, and production of molecules having value as human therapeutic agents. During the past decade, heterocyclic structures received special attention as they belong to a class of compounds with proven utility in medicinal chemistry. There are numerous biologically active bicyclic molecules containing two hetero atoms. 2-Mercaptobenzothiazole (MBT) (**1**, Figure 1) is an important scaffold known to be associated with several biological activities, and its derivatives are manufactured worldwide for a wide variety of applications. S-acethydrazide hydrazone [1] and S-acyl [2] derivatives of MBT were reported to possess antifungal and antibacterial activities, and were also found to be useful in the leather industry [3]. 2-(Thiocyanomethylthio)benzothiazole [4] is a potential contact fungicide for several economically important crops such as barley, cotton, corn, and wheat. 2,2′-Dithiobisbenzothiazole is used as a fungicide, insecticide, sensitizer, and anti-scorching agent in the vulcanization of rubber [5].

A number of methods for the synthesis of 2-mercaptobenzothiazoles (MBTs) have been reported. Among these, classical approaches involve the reaction of thiocarbanilide with sulfur or the interaction of o-aminothiophenol with carbon disulfide under high pressure [6–13]. Several groups reported synthesis of MBTs by the nucleophilic aromatic substitution reaction of a potassium/sodium *o*-ethyl dithiocarbonate with *o*-haloanilines followed by a subsequent cyclization [14–19]. Recently, two efficient approaches from 2-haloaniline precursors were applied for the synthesis of MBTs. The first approach [20] involves a copper-catalyzed condensation reaction of the 2-iodoaniline with thiols in the presence of potassium carbonate. The second [21] involves the reaction of the *o*-haloanilines with carbon disulfide in the presence of 1,8-diazabicyclo[5.4.0]undec-7-ene (DBU).

The last systematic review on the biodegradation and toxicity of MBT was published in 1997 [22], although subsequent surveys have appeared in articles [23, 24] covering the cancer risk from repeated exposure to MBT. Aspects of the chemistry of MBTs have been covered [25–39]. The discovery that MBTs are accelerators of the vulcanization of rubber has stimulated many workers to synthesize and extensively evaluate their derivatives [40–47]. In this review, we present the most significant examples of compounds belonging to this class that exhibit various biological activities reported in literature. The relationship between MBT metal complexes and their pharmacological activities are not included in this review.

## Biologically active 2-mercaptobenzothiazoles

### Antimicrobial activity

2-Mercaptobenzothiazoles exert adverse effects on viruses and also act on yeasts and fungi. The antiviral screening results of MBT showed significant activity against two out of three viruses tested [48]. The anti*-Candida* activity of MBT was studied [49] against 15 *Candida* strains and the results showed 50% growth inhibition at concentrations varying between one and 78 mg L^−1^. The antifungal effects of MBT were also tested against *Aspergillus niger* with a suspension of spore-free mycelium homogenate as inoculum, and a 33 mg L^−1^ MBT concentration was the lower limit for 100% growth inhibition after five days of cultivation. Similar results, although obtained under other conditions, are described [50] for the fungus *Trichophyton rubrum*. It was observed that for complete growth inhibition of *Microsporum gypseum* and *Epidermophyton floccosum,* MBT concentration had to exceed 50 mg L^−1^. The results of a study [51] suggested that the thiol group of MBT is essential for its toxicity, since benzothiazole (BT) was not an active fungicide. However, in another experiment [52] the presence of zinc destroyed the fungicidal activity of MBT, and this contradicts what was suggested above.

**Fig. 2.**
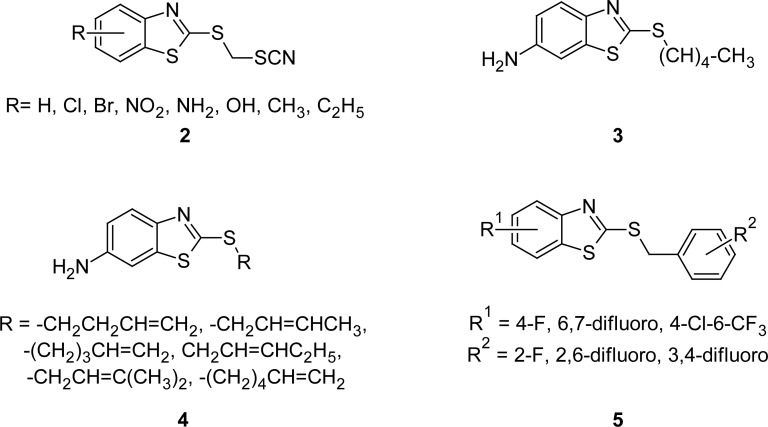
Structure of various congeners of MBT with antifungal activity

The antifungal activity of S-thiocyanomethyl derivatives **2** (Figure 2) of MBT, which in turn were prepared by reacting a metal salt of MBT or substituted MBTs with chloromethyl thiocyanate in an alcohol solution, is described [53]. A significant inhibitory activity against *Aspergillus niger*, *Penicillium roquefortl,* and *Chaetomium globosum* (MIC 75, 50, and 50 ppm, respectively) was observed for the compound 2-(thiocyanomethylthio)benzothiazole (**2**: R=H) after 28 days incubation. On the other hand, 5-chloro and 6-nitro analogues exhibited potent inhibitory activity against *Aspergillus niger* and *Chaetomium globosum* (MIC 5 and 7 ppm, respectively) after 14 days incubation.

The effects of 6-amino-2-*n*-pentylthiobenzothiazole (APB) (**3**, Figure 2) on ergosterol biosynthesis in *Candida albicans* and *Saccharomyces cerevisiae* were studied [54–57]. Authors disclosed that APB markedly blocks formation of ergosterol in *Saccharomyces cerevisiae,* and the accumulation of squalene, lanosterol, 4-methylzymosterol, and 4,4-dimethylzymosterol were observed. In search of potent antifungal agents, 2-(alkenylthio)-5-aminobenzothiazoles **4** (Figure 2) were synthesized and screened for their antimicrobial activity [58–60]. Authors disclosed antibacterial activity of the tested compounds against *Staphylococcus aureus* and anticandidous activity against *Candida albicans*. Compounds bearing –(CH_2_)_3_-CH=CH_2_ or –CH_2_CH=CH-C_2_H_5_ groups at second position of the MBT ring showed maximum inhibitory effect (MIC 15.6 μg mL^−1^) against *Candida albicans*. Various derivatives of 2-alkylthio-6-aminobenzothiazoles have also shown significant anti-yeast activity [61, 62]. Moreover, their formamido and benzamido derivatives have manifested antimycobacterial [63, 64], antifungal [65], and anti-algal [66] activities. In another study, a series of polyfluorinated 2-benzylthiobenzothiazoles **5** (Figure 2) were prepared [67] and tested for their antifungal activities. Most of the synthesized compounds showed significant fungicidal activity against *Rizoctonia solani*, *Botrytis cinereapers,* and *Dothiorella gregaria* at 50 μg mL^−1^concentration.

**Fig. 3.**
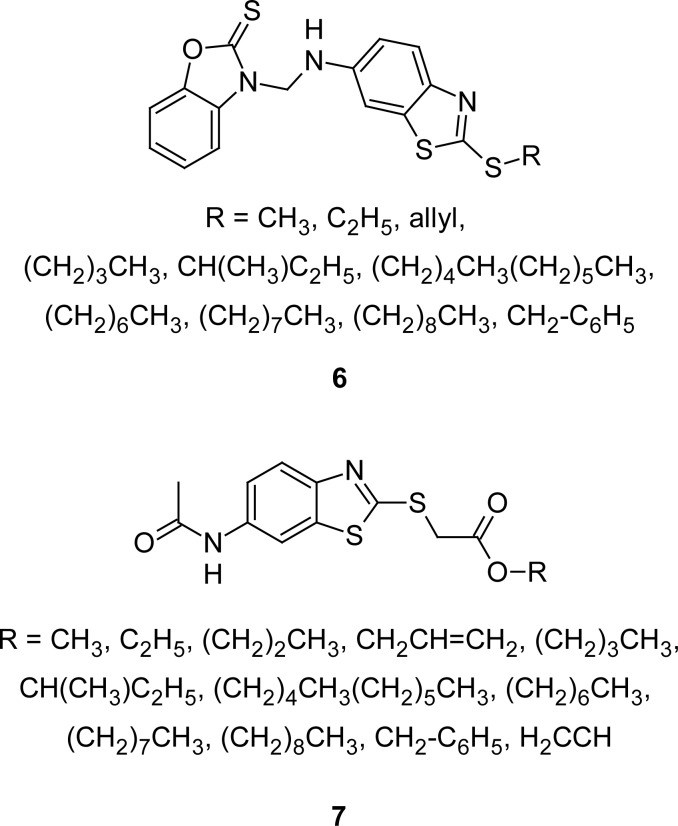
Analogues of MBT that have shown inhibition of oxygen evolution in spinach chloroplasts

In a separate communication [68], an author disclosed the anti-candida activity of 3-(2-alkylsulfanyl-6-benzothiazolylaminomethyl)-2-benzothiazolethiones, however compounds were not stable enough when stored for a longer period of time. Replacement of one sulfur atom in the heterocyclic system by an oxygen atom solved the stability of products, and various benzoxazolethiones **6** (Figure 3) were prepared [69, 70] by the reaction of 2-alkylsulfanyl-6-aminobenzothiazoles with 3-hydroxymethyl-2-benzoxazolethione in ethanol. A derivative having a benzyl group at second position of the MBT ring showed maximum inhibition of the oxygen evolution rate in spinach chloroplasts. Photosynthetic-inhibiting activity of a novel series of 2-(6-acetamidobenzothiazolethio)acetic acid esters **7** (Figure 3) was also reported [71]. Compounds were synthesized by acetylation of 2-(alkoxycarbonylmethylthio)-6-aminobenzothiazoles with acetic anhydride. Compounds having a hexyl acetate group on the second position of the MBT ring exhibited maximum inhibition (IC_50_ 47 μmol dm^−3^) of the oxygen evolution rate in spinach chloroplasts.

MBT also exerts adverse effects on bacteria and for this reason, the compound was under investigation as a potential nitrification inhibitor in soils [72]. Another example of the antibacterial activity of MBT is given [50] and it was considered to have a strong inhibitory effect on *Mycobacterium tuberculosis*[73–75]. Extensive studies [76] clearly indicated that MBT inhibited the growth of bacteria and yeast, but its effects were bacteriostatic rather than bactericidal, and were different for different species. MBT was investigated [77] as an inhibitor of dopamine β-hydroxylase, which converts dopamine into the neurotransmitter noradrenaline. In some reports, the effect of MBT on specific bacterial enzymes is also studied [78, 79].

**Fig. 4.**
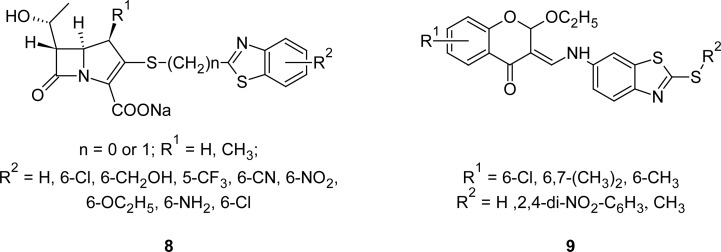
Analogues of MBT linked to carbapenem and chroman-4-one with promising antibacterial activity

MBT derivatives have been pointed to as promising antibacterial agents. For example, the benzothiazol moiety linked by sulfur to the 3-position of carbapenems **8** (Figure 4) displayed significant potency against methicillin-resistant *Staphylococcus aureus* (MRSA) [80], leading to the hypothesis that the 2-mercaptobenzothiazole moiety is a “binding element”. It was observed that the introduction of a 1-β-methyl group at position one enhanced the human renal dehydropeptidase-1 (DHP-1) stability of compounds at least six-fold more compared to the 1-β-hydrogen analogues. Further, the introduction of a 1-β-methyl group appeared to enhance potency against MRSA.

**Fig. 5.**
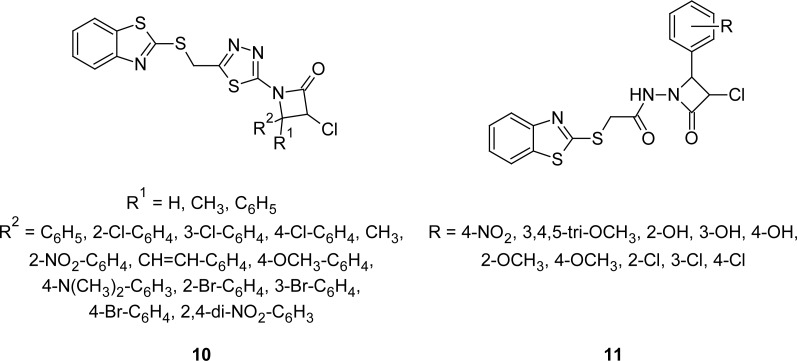
Analogues of MBT linked to 1,3,4-thiadiazol and azetidinone moieties with antibacterial activity

A series of compounds **9** (Figure 4) containing a MBT nucleus linked to the chroman-4-one moiety have been prepared and evaluated for their antimicrobial activities [81]. Derivatives bearing 6-Cl or 6,7-dimethyl substituents on the chroman-4-one moiety exhibited significant activity against the Gram-positive bacteria *Staphylococcus aureus* and *Bacillus subtilis,* and against *Mycobacterium tuberculosis*. The same compounds showed potent activity against *Candida albicans* and *Saccharomyces cerevisiae*. MBT derivatives **10 (**Figure 5) possessing both 1,3,4-thiadiazole and 3-chloro-2-azetidinone moieties have also been reported to possess antibacterial activities [82, 83]. Derivatives having phenyl or 4-chlorophenyl substituents at fourth position of the azetidinone nucleus were found to be the most potent compounds against both Gram-positive and Gram-negative bacterial strains.

Novel 4-substituted phenyl-3-chloro-1-[(benzothiazolylthio)acetamidyl]-2-azetidinones **11** (Figure 5) have been synthesized by both conventional and microwave methods [84]. The synthesized compounds were screened for their antibacterial and antifungal activities by the paper disc diffusion method. Compounds having 4-OH-C_6_H_4_ and 2-Cl-C_6_H_4_ groups at fourth position of the azetidinone nucleus were most active (inhibition zone 16–22 mm) against the tested Gram-positive bacteria *Staphylococcus aureus* and fungus *Aspergillus niger,* whereas compounds possessing 3-OH-C_6_H_4_ and 3-Cl-C_6_H_4_ groups exhibited significant activity (inhibition zone 16–22 mm) against *Candida albicans*.

**Fig. 6.**
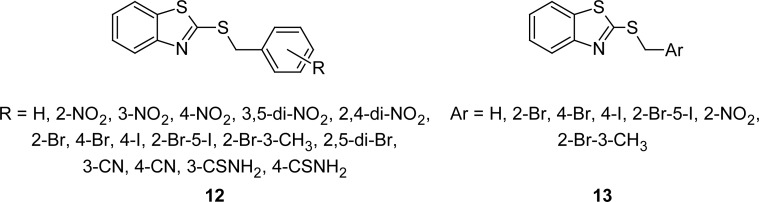
Analogues of MBT linked to a benzyl moiety with antibacterial activity

2-Benzylsulfanyl derivatives **12** (Figure 6) of MBT have been synthesized by the nucleophilic substitution reaction of the sodium salt of MBT upon benzyl halide in *N*,*N*-dimethylformamide at room temperature. The derivative bearing a CN group was further converted into a thioamide through the reaction with hydrogen sulfide in pyridine in the presence of triethylamine [85]. Compounds bearing 3,5-dinitro or 2,4-dinitro groups on the benzene ring exhibited significant activity against *Mycobacterium kansasii* 235/80 (MIC 2–4 μmol L^−1^) and exceeded the activity of isoniazid (INH) (MIC = > 250 μmol L^−1^), however compounds showed only weak activity (MIC 8 μmol L^−1^) against *Mycobacterium tuberculosis* 331/88, and the activity of INH was not reached (MIC 0.5–1 μmol L^−1^). The substitution of a thioamide group either on third or fourth position on the benzene ring afforded potent compounds, but they were less active compared to the dinitro derivatives.

In a separate communication, the synthesis and antimicrobial activities of 2-benzylsulfanyl derivatives **13** (Figure 6) of MBT are also described. Compounds were synthesized [86] in 94–98% yields by the reaction of MBT and benzyl bromide in refluxing acetone, in the presence of K_2_CO_3_. Synthesized compounds were found to be either weakly active or inactive against *Escherichia coli*. All of the tested compounds showed no activity against *Bacillus subtilis*, *Micrococcus luteus,* and *Pseudomonas aeruginosa*. Tested compounds were also found to be either weakly active or inactive against *Candida albicans* and *Aspergillus niger*.

**Fig. 7.**
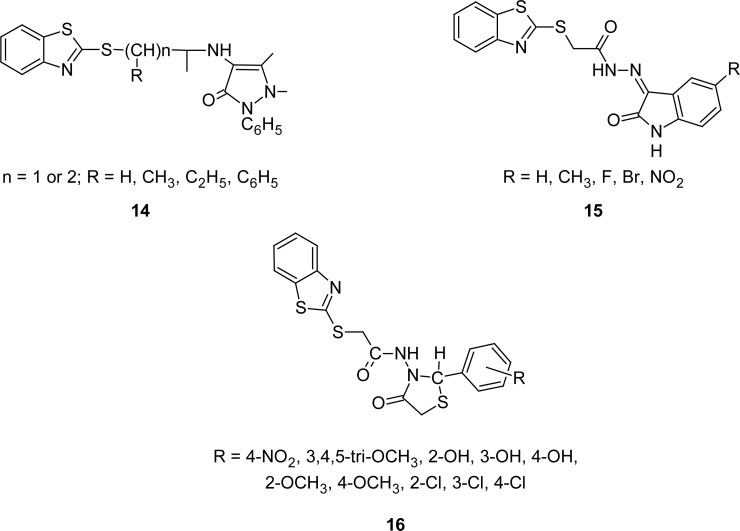
Structure of various congeners of MBT that have shown antibacterial property

Novel MBT derivatives bearing pyrazolone **14** and indolinone moieties **15** (Figure 7) at second position of the MBT nucleus have been synthesized. Synthesized compounds were evaluated for their antimicrobial activities using the disc diffusion method [87]. Derivatives belonging to the indolinone series **15** were found to be more active compared to the pyrazolone series **14** against *Staphylococcus epidermidis*. Moreover, derivatives bearing nitro or fluoro groups at fifth position of the indolinone nucleus exhibited maximum activity against *Staphyloccocus epidermidis*.

A series of compounds **16** (Figure 7) containing a MBT nucleus linked to the 1,3-thiazolidine-4-one moiety through a acetamido group have been synthesized by both microwave and conventional methods using ZnCl_2_ as a catalyst. The synthesized compounds were screened for their *in vitro* antimicrobial activities [88]. Screening results revealed significant inhibitory activity (inhibition zone 20–25 mm) of derivatives having a 2-OCH_3_-C_6_H_4_ group at second position of the thiazolidine ring against *Escherichia coli*, *Candida albicans,* and *Candida parapsilosis*, whereas derivatives bearing 4-NO_2_-C_6_H_4_, 2-OH-C_6_H_4_, 4-OH-C_6_H_4_, 4-OCH_3_-C_6_H_4_, 2-Cl-C_6_H_4,_ and 4-Cl-C_6_H_4_ groups were found to have moderate activity (inhibition zone 15–20 mm) against *Bacillus subtilis*, *Staphylococcus aureus,* and *Escherichia coli*. The rest of the tested compounds were found to be either weakly active or inactive. On the other hand, derivatives bearing 4-NO_2_-C_6_H_4_, 4-OH-C_6_H_4_, 4-OCH_3_-C_6_H_4,_ and 4-Cl-C_6_H_4_ groups also exhibited moderate inhibitory activity (inhibition zone 15–20 mm) against *Candida albicans*, *Candida krusei,* and *Candida parapsilosis*.

### Anti-inflammatory activity

**Fig. 8.**
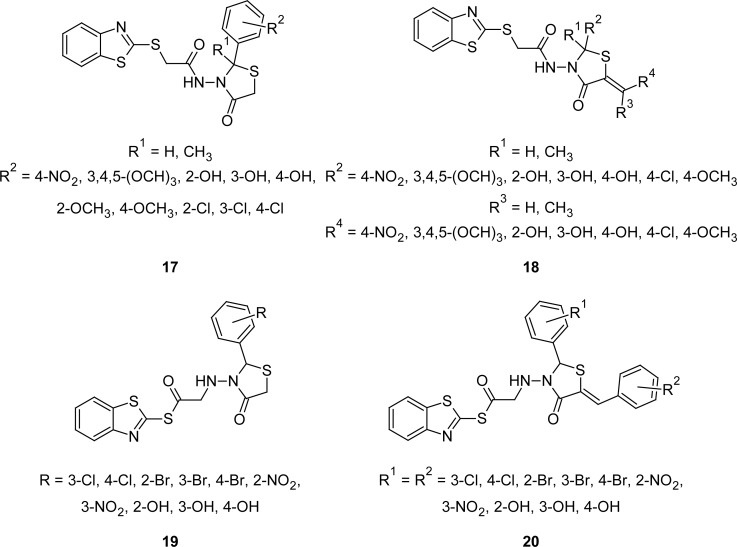
1,3-Thiazolidine-4-one incorporated MBTs that have shown anti-inflammatory activity

MBT is the important pharmacodynamic heterocyclic nucleus, which when incorporated in different heterocyclic templates has been reported to possess potent anti-inflammatory activity. For example, MBT derivatives possessing 4-oxothiazolidines **17** and their 5-arylidenes **18** (Figure 8), showed moderate to weak anti-inflammatory activity in the carrageenan-induced paw edema in rats [89] at an oral dose of 50 mg kg^−1^ body weight. Three compounds (**17**: R^1^ = H, R^2^ = 3-Cl; **18**: R^1^ = CH_3_, R^2^ = 4-Cl-C_6_H_4_, R^3^ = CH_3_, R^4^ = 4-Cl-C_6_H_4_ and **18**: R^1^ = H, R^2^ = 4-Cl-C_6_H_4_, R^3^ = H, R^4^ = 4-Cl-C_6_H_4_) showed significant protection (38.8–44.4%) against carrageenan -induced edema, while two compounds (**17**: R^1^ = CH_3_, R^2^ = 4-CH_3_O; **18**: R^1^ = CH_3_, R^2^ = 2,4-(NO_2_)_2_-C_6_H_3_, R^3^ = CH_3_, R^4^ = 2,4-(NO_2_)_2_-C_6_H_3_ were found to be inactive. Few compounds also showed significant inhibitory activity against *Bacillus subtilis*, *Escherichia coli,* and *Salmonella typhimurium*. The synthesized compounds were also evaluated against the experimental infection *Ancyclostoma ceylanicum* in hamsters and *Hymenolepsis nana* in rats at a dose of 250 mg kg^−1^. Few compounds cleared 100% of the *Hymenolepsis nana* infection and showed significant activity against *Ancyclostoma ceylanicum*.

In a similar work, novel 1,3-thiazolidin-4-ones **19** and their 5-arylidenes **20** (Figure 8) have been synthesized and evaluated for their antimicrobial activity by the paper disc diffusion method. Compounds were also evaluated for their anti-inflammatory activity by the carrageenan-induced paw edema in rats [90]. Authors disclosed significant antibacterial and antifungal activities for a few compounds, but no appreciable increase in the anti-inflammatory activity was observed.

**Fig. 9.**
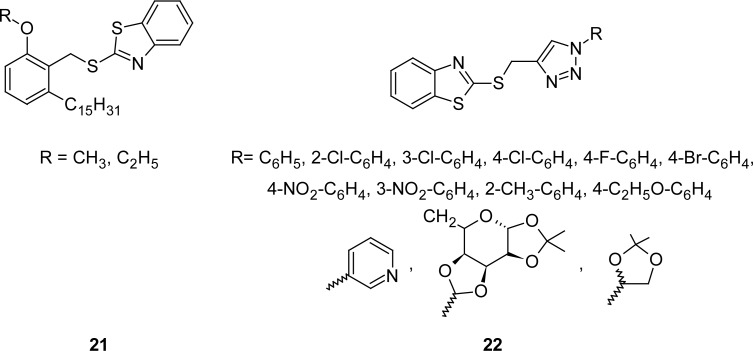
Structure of MBTs that have shown promising COX-2 inhibitory activity

In attempt to improve the potency and selectivity towards the cyclooxygenase-2 (COX-2) enzyme, 2-{[2-alkoxy-6-pentadecylphenyl)methyl]thio]-1*H*-benzothiazoles **21** (Figure 9) has been synthesized [91] by reaction of 2-alkoxy-6-pentadecylbenzyl alcohol with thionyl chloride followed by the condensation with MBT in the presence of tetrabutyl ammonium bromide. The synthesized compounds were screened for their human COX-2 inhibitory activity. The compound bearing a OCH_3_ group at the second position of the phenyl ring was found to be 470-fold more selective towards COX-2 compared to COX-1.

Recently, novel bis-heterocycles **22** (Figure 9) encompassing 2-mercaptobenzothiazole and 1,2,3-triazoles were synthesized [92] by the cycloaddition reaction of 2-(prop-2-ynylthio)benzo[*d*]thiazole with various azides. The synthesized compounds were evaluated for their anti-inflammatory activity by the carrageenan-induced hind paw edema in rats. The structure-activity relationship revealed that the aromatic ring attached to the triazollyl moiety is essential for potential activity when compared to aliphatic/alicyclic rings. Electron withdrawing groups, when substituted on an aromatic ring mostly at the para position, exhibited potent anti-inflammatory activity compared to the standard drug ibuprofen, without causing any ulceration. The COX-2 inhibitory potential of compounds having a 4-fluorophenyl group at first position of the triazole ring and ulcerogenic studies further conclude that these kinds of molecules can be considered as potent anti-inflammatory agents.

**Fig. 10.**
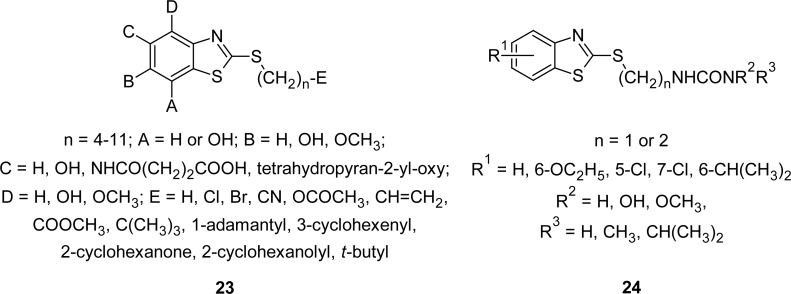
Structure of MBTs that have shown promising lipoxygenase inhibitory activity

A series of MBTs **23** (Figure 10) has been synthesized [93] by the reactions of substituted 2-mercaptobenzothiazole with the appropriate bromo derivative in a basic solution, e.g., dimethylformamide/triethylamine; dimethylsulfoxide/triethylamine; or 1N sodium hydroxide/tetrahydrofuran. Compounds were identified as inhibitors of leukotriene biosynthesis and/or as inhibitors of the action of lipoxygenase. Several of the compounds were also tested for their inhibitory effect on mucous secretion in the canine tracheal secretion model. The 6-{[-[(3-carboxy-1-oxopropyl)amino]-2-benzothiazolyl]thio}hexanoic acid methyl ester caused marked inhibition of mucous secretion.

In another study, a novel series of *N*-[(2-benzothiazolylthio)alkyl]-*N*′-urea derivatives **24** (Figure 10) were synthesized and evaluated both *in vivo* and *in vitro* for their 5-lipoxygenase inhibitory activity [94]. Compounds were prepared by the sequential treatment of 3-(2-benzothiazolylthio)carboxylic acids with oxalyl chloride and the appropriate amine nucleophiles. It was observed that the hydroxyl group of the hydroxyurea must be unsubstituted in order to be active. N′-methyl substituents imparted superior activity *in vivo,* however, this correlation was not as pronounced *in vitro*. A three carbon chain was found to be optimum for activity. In general, the compounds were surprisingly sensitive to position and nature of the substituent R^1^ on the benzo portion of the benzothiazole nucleus. The 5-chloro substituent present in the benzo portion markedly enhanced the activity both *in vitro* and *in vivo,* however, the 7-chloro analogue showed diminished activity *in vitro* and was inactive *in vivo*. Replacing the 5-chloro substituent with a 5-trifluoromethyl caused a > 40-fold loss of activity *in vitro* and virtually a total loss of activity *in vivo*. Other substitutions such as 6-ethoxy and 6-isopropyl also resulted in inferior activity relative to the unsubstituted analogues.

**Fig. 11.**
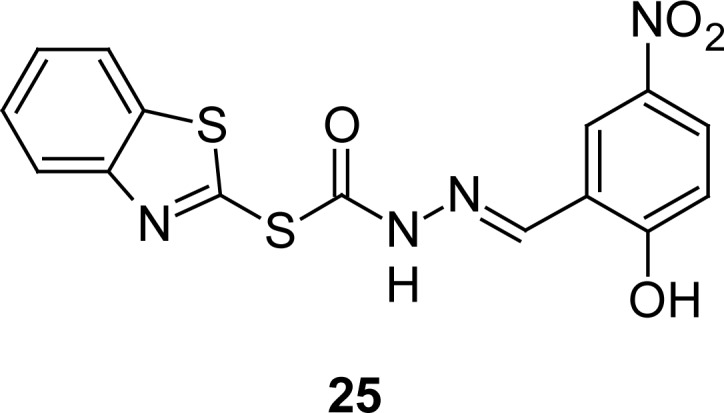
Structure of compounds that have shown selective inhibition of neutrophil activation by inflammatory mediators

In search of compounds that can selectively inhibit neutrophil activation by inflammatory mediators, compound **25** (Figure 11) was identified [95] by means of high throughput, which inhibited the respiratory burst (RB) of human neutrophils in response to soluble inflammatory mediators, such as TNF and formylated methionyl-leucyl-phenylalanine (fMLF), but not in response to phobol myristate acetate (PMA), and did not suppress the antibacterial activity of neutrophils.

**Fig. 12.**
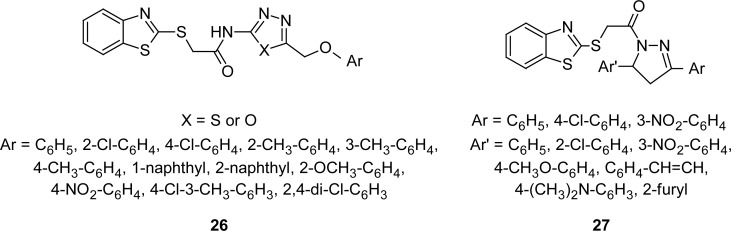
Structure of dual-acting antibacterial-anti-inflammatory MBTs

In an attempt to develop dual-acting antimicrobial and analgesic-anti-inflammatory agents, 1,3,4-oxadiazole/1,3,4-thiadiazole (**26**) and 2-pyrazoline (**27**) incorporated MBTs (Figure 12), were synthesized [96–98]. Some of these compounds showed specific activity against the Gram-negative bacteria *Pseudomonas aeruginosa*. Among synthesized compounds, the 1,3,4-oxadiazole derivative having 4-nitrophenyl group group at fifth position of the oxadiazole ring, the thiadiazole derivative having 2-methylphenyloxymethyl group at fifth position of the thiadiazole ring, and the pyrazoline derivative having 3-nitrophenyl and phenyl groups, respectively, at third and fifth position of the pyrazoline ring exhibited significant protection (76.9–81.6%) against carrageenan-induced paw edema in rats with a significant reduction in gastrointestinal toxicity.

**Fig. 13.**
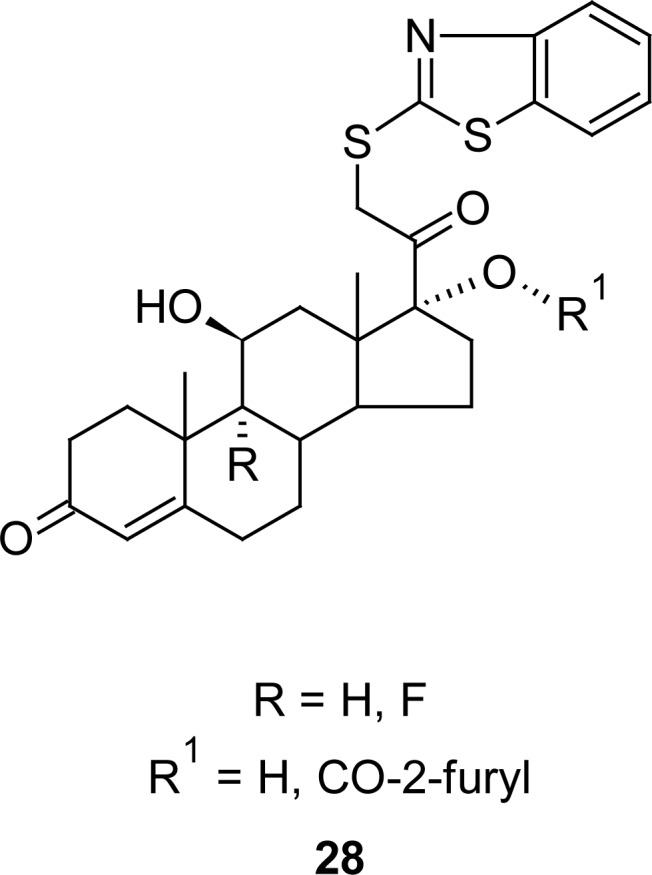
Analogues of MBT that showed excellent dissociated glucocorticoid property

In an effort for the discovery and development of dissociated glucocorticoids for the treatment of various inflammatory diseases, C-21 MBTs **28** (Figure13) were prepared [99] by the treatment of hydrocortisone mesylate with MBT in acetone. A compound possessing furan-2-carboxylate group at seventeenth position of the steroidal nucleus (**28** R = H and R^1^ = CO-2-furyl) showed a significant dissociated profile in *in vitro* functional assays, but also a pharmacological profile in a Brown-Norway rat therapeutic index model of asthma that dissociated side effects (thymolysis), while maintaining efficacy against pulmonary inflammation and lung function. The results showed an IL-6 inhibition with an IC_50_ of 2 nM compared to the desfuroate compound (R and R^1^ = H; IC_50_ 36 nM). In the nose-only inhalation experiment, the micronized compound **28** (R = H and R^1^ = CO-2-furyl) demonstrated dose-dependent improvement in lung function with an ED_70_ of 47 μg kg^−1^ daily estimated pulmonary deposition. Moreover, this compound failed to induce thymolysis ≥ 20% at any dose tested up to 730 μg kg^−1^ and the therapeutic index was found to be greater than other compounds compared to the positive control fluticasone propionate.

### Anthelmintic activity

**Fig. 14.**
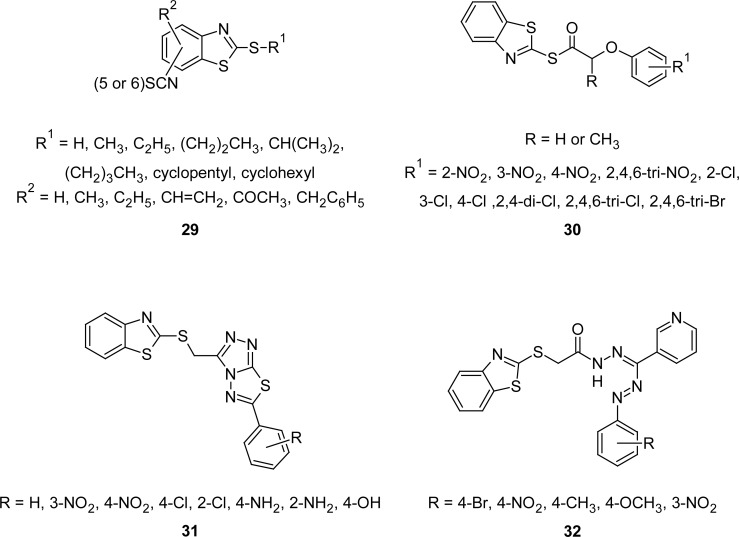
Various congeners of MBT that have shown promising anthelmintic property

2-Mercaptobenzothiazoles have been pointed to as promising anthelmintic agents. For example, 2-alkylthio-5/6-isothiocyanobenzothiazoles **29** (Figure 14) were found to be effective anthelmintics [100] when tested in mice infested with *Hymenolepsis nana* or *Nematospiroides dubius*, in rats infested with *Fasciola hepatica,* and in fowl infested with *Ascaridia galli*. The same compounds were also tested for their antimicrobial activities against different strains of Gram-positive and Gram-negative bacteria and against ten strains of fungi. Some of the tested compounds exhibited significant anthelmintic and antibacterial activities.

A series of novel, substituted phenoxyacetyl as well as propionyl-2-mercaptobenzothiazoles **30** (Figure 14) were synthesized [101] by reaction of aryloxyacetyl chloride with an ice cold alkaline solution of MBT in acetone. Synthesized compounds were tested for their anthelmintic, analgesic, and antimicrobial activities. Compounds having 2,4,6-trichloro or 2,4,6-tribromo groups in the phenyl ring and propionyl group as a linker between aryloxy and the MBT ring cleared 40–70% of worms in hamsters infested with *Ancyclostoma ceylanicum* and *Nipostrongylus brasiliensis.* The same compounds also showed significant analgesic activity using the tail flick method in rats. Some of the compounds, particularly di and trihalo derivatives, showed marked inhibitory activity (MIC 1–2.5 μg mL^−1^) against *Bacillus anthracis* and *Candida albicans*.

Derivatives of MBT containing [1,2,4]triazolo[3,4-*b*][1,3,4]thiadiazoles **31** and substituted phenyldiazenyles **32** (Figure 14) moieties were also found to be effective against *hymenolepis nana* infection in mice [102, 103]. Among synthesized [1,2,4]triazolo– [3,4-*b*][1,3,4]thiadiazoles **31**, maximum activity (60% at a dose of 250 mg kg^−1^ orally) was observed in compounds having 3-NO_2_-C_6_H_4_ or 4-Cl-C_6_H_4_ groups at fifth position of the thiadiazole nucleus, whereas substitution of 4-NO_2_-C_6_H_4_ or 4-OH-C_6_H_4_ groups markedly reduced the activity. It was also observed that substitution of 2-Cl-C_6_H_4_, 2-NH_2_-C_6_H_4_, or 4-NH_2_-C_6_H_4_ groups resulted in complete loss of activity. While among substituted phenyldiazenyles **32**, maximum anthelmintic activity (60%) was observed in compounds with methoxy and chloro substituents at fourth position in the phenylazo moiety. Compounds with 4-nitro and 4-methyl substituents in the same moiety showed 40% activity.

### Miscellaneous

**Fig. 15.**
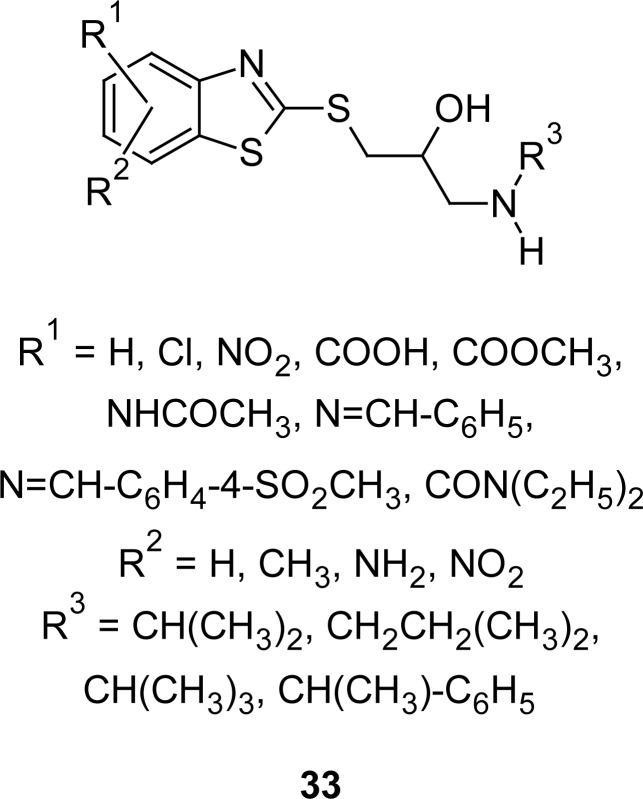
Novel series of MBT derivatives having antihypertensive property

With the aim of developing novel compounds with improved potential for treating hypertension, propanolamine analogues of MBT **33** (Figure 15) were investigated [104]. Compounds were synthesized by the reaction of aminoalchohol with MBT, or substituted MBT in organic solvent in the presence of base below 30 °C, under nitrogen. Most of the compounds exhibited significant prolonged antihypertensive effect without noticeable β-adrenergic receptor blocking activity.

**Fig. 16.**
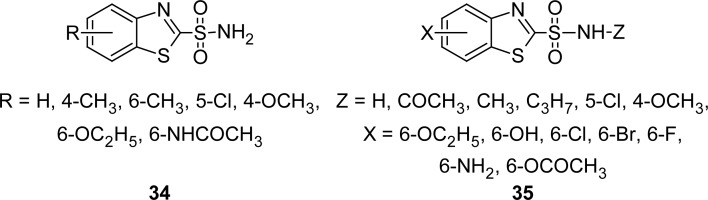
Two novel series of MBT active against the enzyme carbonic anhydrase

In the year 1973, carbonic anhydrase inhibitory activity of a number of aryl-substituted benzothiazole-2-sulfonamides **34** (Figure 16) was described [105]. All of the tested compounds showed potent carbonic anhydrase inhibitory activity and one of these, the 6-ethoxybenzothiazole-2-sulfonamide, produced a clinically useful diuresis. In another report, synthesis of analogues of benzothiazole-2-sulfonamides **35** (Figure 16) as carbonic anhydrase inhibitors was described [106]. Compounds were tested topically for their ability to reduce intraocular eye pressure in glaucoma. Among these, 6-chlorobenzothiazole-2-sulfonamide was found to be highly effective.

**Fig. 17.**
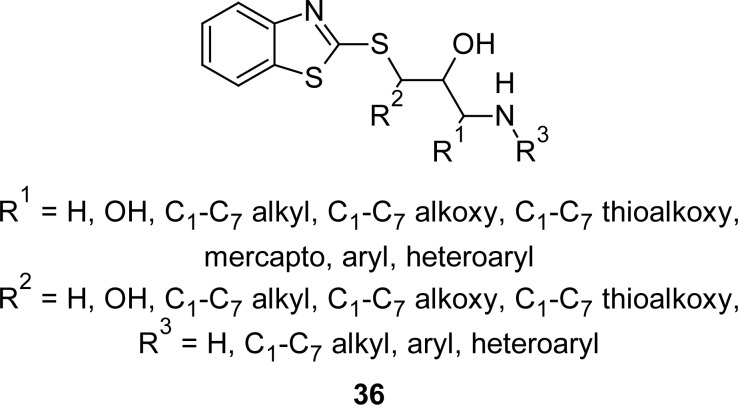
Analogues of MBT that have shown high affinity for GABA_B_ receptor

Analogues of MBT **36** (Figure 17) containing a substituted hydroxypropyl carbamate group at the second position have been synthesized [107] and evaluated for the inhibition of transient lower esophageal sphincter relaxations, and for the treatment of gastro-esophageal reflux disease, and found to have high affinity and potency for the GABA_B_ receptors as revealed by low IC_50_ and EC_50_ in the ^3^H GABA radioligand binding and ileum assay, respectively.

**Fig. 18.**
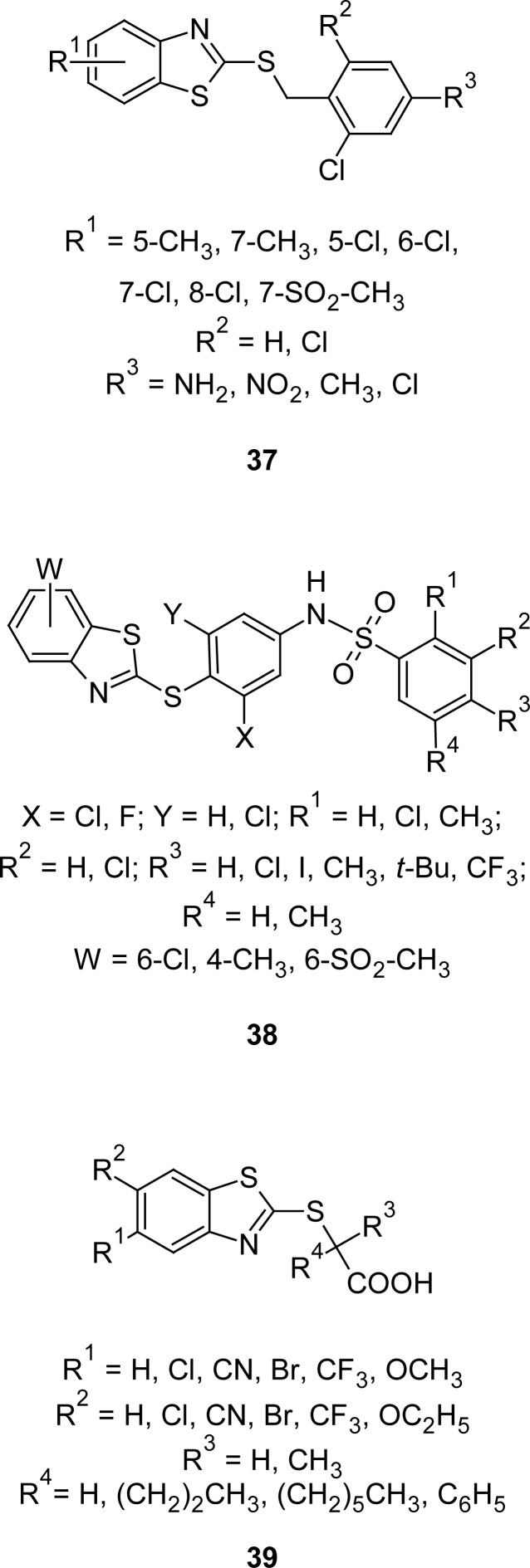
Various congeners of MBT having PPARγ receptor activator property

In another study, MBT analogues **37** and **38** (Figure 18) have been synthesized and evaluated [108] for their ability to displace radiolabeled rosiglitazone (BRL 49653) from a peroxisome proliferator-activated receptor glutathione-S-transferase (PPARγ-GST) fusion protein. Compounds were identified as PPARγ activators and found to be useful agents for the treatment of obesity and related disorders associated with undesirable adipocyte maturation.

In 2009, several 2-benzothiazolylthioalkanoic acids **39** (Figure 18) were synthesized and evaluated for their human PPARγ transactivation activity [109]. Overall, the potencies of some newly designed agonists were slightly higher than those of typical fibrates, such as clofibrate. While unsubstituted derivatives proved inactive, the overall effect of the introduction of substituents in the 5 and 6 positions improved PPARα agonistic activity. Among the synthesized compounds, the 5-bromo derivative (R^1^ = Br, R^2^ = H, R^3^ and R^4^ = CH_3_) with an EC_50_ value of 2.5 μmol was found to be 10–20 times more effective than other compounds of the same series.

**Fig. 19.**
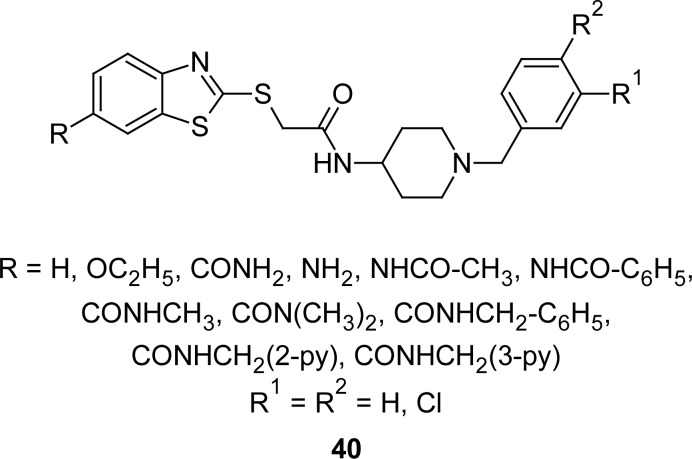
Analogues of MBT that have shown the CCR3 receptor antagonist property

In search of an orally active non-peptide antagonist selective for the CCR3 receptors, the 2-(benzothiazolylthio)acetamide derivative **40** (R = R^1^ = R^2^ =H, Figure 19) was identified as the leading compound which on further derivatization led to the identification of potent and selective antagonists [110, 111]. The 7-acetamidobenzothiazole derivative **40** (R = –NHCOCH_3_, R^1^ = R^2^ = Cl) with an IC_50_ of 1.5 nM and a 3600-fold selectivity over that of the CCR1 receptor was found to be the best compound of this series.

**Fig. 20.**
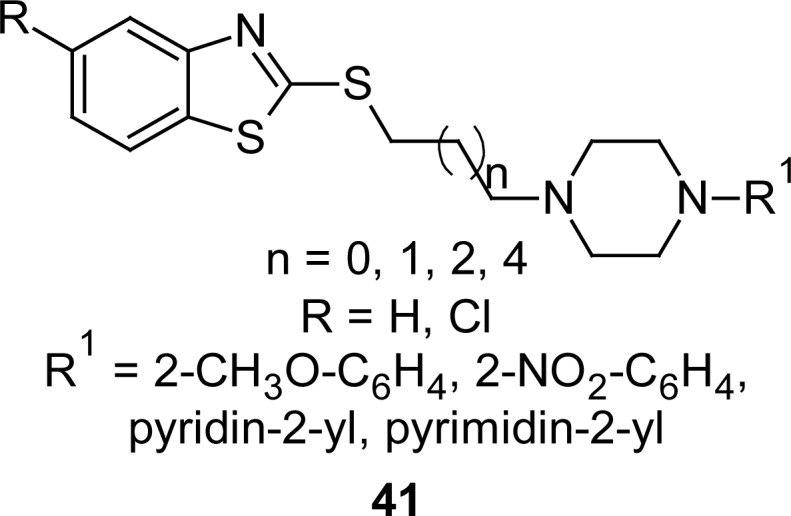
Analogues of MBT with high affinity for 5-HT_1A_ receptors

A series of compounds **41** (Figure 20) containing a MBT nucleus linked at the second position to an arylpiperazine by different alkyl chains, were prepared [112] by reacting the appropriate 1-ω-chloroalkyl-4-arylpiperazine with MBT or 6-chloro-2-mercaptobenzothiazole in acetone at reflux, in the presence of potassium carbonate and potassium iodide. They were tested in radioligand binding experiments to evaluate their affinity for 5-HT_1A_ and 5-HT_2A_ receptors. Compounds possessing a 2-nitrophenylpiperazine group showed lower affinity for the 5-HT_1A_ receptor with respect to the 2-methoxyphenylpiperazine analogues. In addition, compounds characterized by a propyl side chain between the terminal fragment and the arylpiperazine portion exhibited higher affinity toward 5-HT_1A_ with respect to the analogues containing an ethyl chain as linker. In particular, among propyl derivatives, compounds possessing 2-methoxyphenylpiperazine showed an affinity for the 5-HT_1A_ receptor (R) in the subnanomolar range (*K_i_* 0.29 nM), coupled to a high selectivity over α_1_-adrenergic receptors (*K_i_* α_1_-adrenergic R/*K_i_* 5-HT_1A_ R=114). Recently, another group of researchers investigated the binding affinities of these compounds **41** to 5-HT_1A_ and α_1_-adrenergic receptors in terms of structural requirements [113]. The binding affinity was found to be the function of the cumulative effect of different structural features which were identified in terms of individual descriptors.

**Fig. 21.**
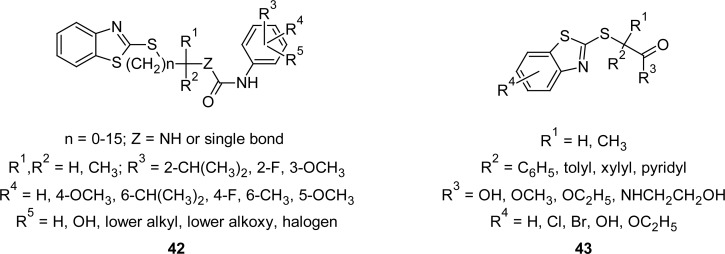
Various congeners of MBT having ACAT inhibitory property

A new series of pharmacologically active anilides **42** (Figure 21) is described in literature. The synthesis was carried out by reaction of a carboxylic acid or its reactive derivative with an aniline derivative to give an amide derivative, which on further treatment with MBT gave anilides **42**. Authors disclosed acyl coenzyme A cholesterol acyltransferase (ACAT) inhibition activity in rabbits, which is therefore useful as an anti-hyperlipidemic agent [114, 115]. Apart from acyl coenzyme A cholesterol acyltransferase (ACAT) inhibition activity, serum cholesterol and triglyceride lowering activities are also reported [116] to be associated with MBT derivatives **43** (Figure 21). A shift in the ratio of α-lipoproteins and β-lipoproteins in the direction of increasing α-lipoproteins was also observed.

**Fig. 22.**
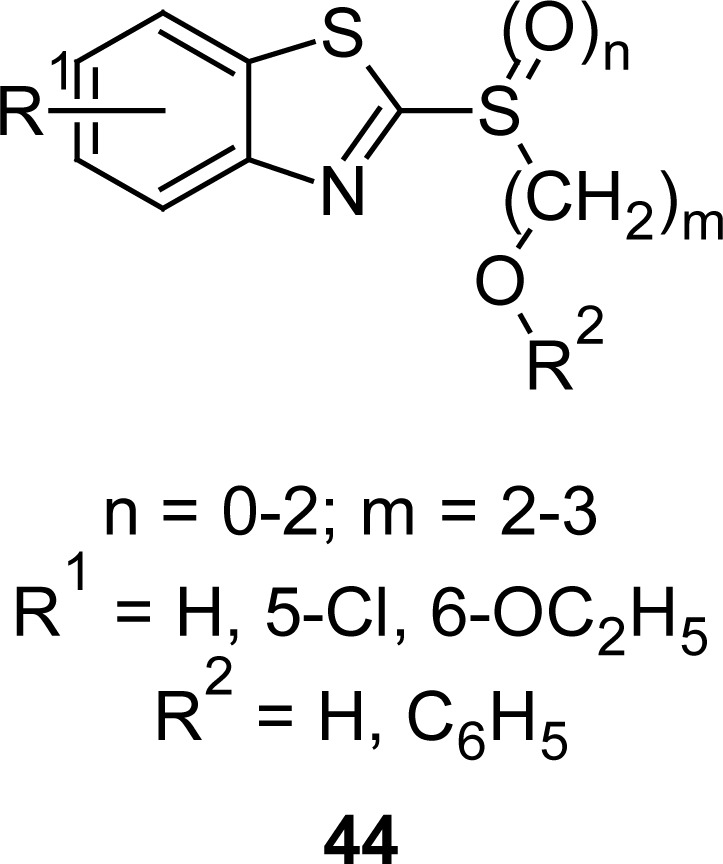
Analogues of MBT having promising antiulcer property

2-Mercaptobenzothiazoles are also indentified as antiulcer agents. For example, synthesis and antiulcer activity of 5-chloro-2-[(2-alkoxy-ethyl)thio/sulfanyl/sulfonyl]benzothiazoles **44** (Figure 22) is described in literature [117]. The test compound 5-chloro-2-[(2-ethoxyethyl) sulfanyl]benzothiazole exhibited a potent ulceration inhibitory ratio in the submerged restraint stress, histamine-induced, and aspirin-induced ulcer tests (93.0, 50.0, and 85.0%, respectively) compared to the standard drug omeprazole (99.0% in each case).

**Fig. 23.**
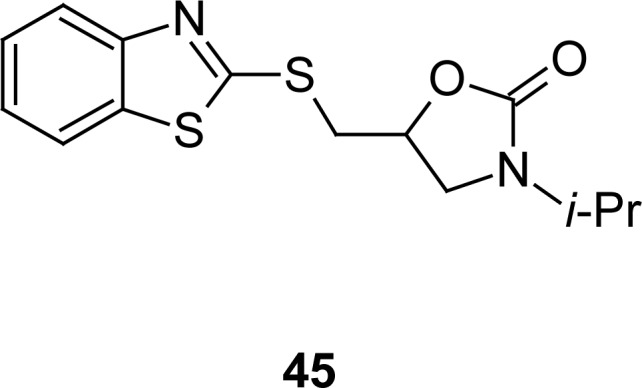
Analoges of MBT that have shown MAO inhibitory property

Selective monoamine oxidase (MAO) inhibitors have been developed to treat a variety of neurological disorders. In this regard, attention was focused on the incorporation of the 3-*iso*-propyloxazolidin-2-one moiety at second position of the MBT nucleus through the methylene group, and these enantiomerically pure and/or racemic form molecules (**45**) were biologically tested for MAO-A and MAO-B activities by bovine mitochondria as the enzyme source and kynuramine as the substrate. The racemic mixture and enantiomers of compound **45** (Figure 23) were found to be equipotent with no MAO-A/MAO-B selectivity [118].

**Fig. 24.**
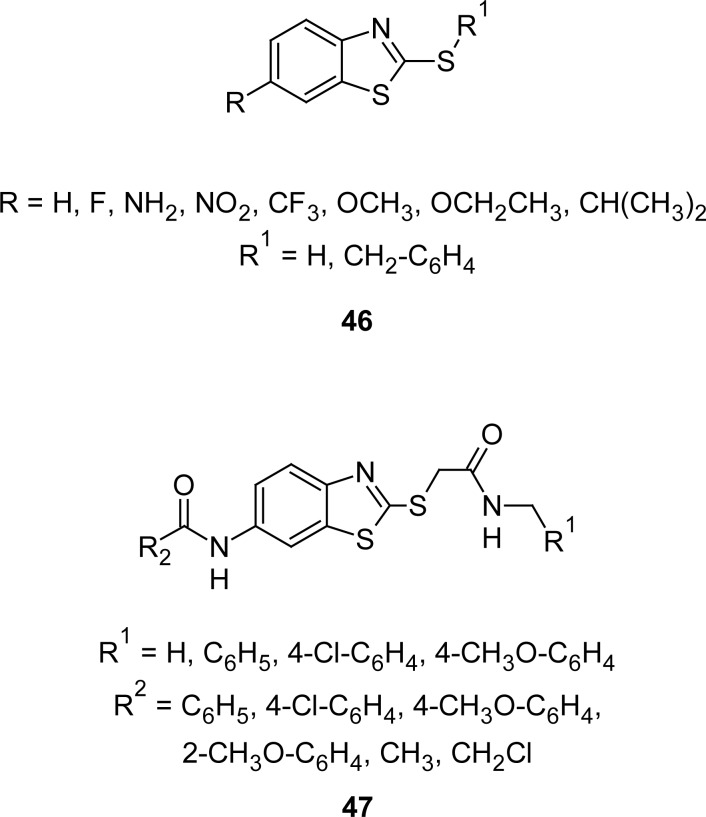
Various congeners of MBT having promising antitumor activity

MBTderivatives are capable of suppressing the *in vitro* growth of various types of tumor cells. Novel 2-benzylthio-6-substituted-1,3-benzothiazoles **46** (Figure 24) were synthesized [119] by the interaction of appropriate aniline with potassium ethyl xanthate in DMF and the resultant 6-substituted-1,3-benzothiazole-2-thiols were further treated with benzyl bromide and K_2_CO_3_ in a mixture of dioxane/water. Synthesized compounds were assayed against human cervical cancer (HeLa) and normal human lung fibroblast (MRC-5) cell lines, following a three-days exposure. In general, the cancer cells were more sensitive to the tested agents. The derivatives bearing the 6-CF_3_ or 6-NO_2_ group tested at the single dose of 100 μM, did not produce a relevant change in cell viability in MRC-5 cells, whereas their cytotoxic effect in HeLa cells was remarkable (about 80% of inhibition). Antimicrobial screening results showed significant inhibitory activity for compounds **46** (R_1_=H) against the tested bacterial strains with MIC values between 3.12 and 100 μg mL^−1^. In this regard, compounds having CF_3_ and NO_2_ groups at sixth position of the MBT ring showed promising results, and in particular, the compound with a 6-CF_3_ group exhibited maximum activity (MIC 3.12 μg mL^−1^) against *Staphylococcus aureus*. On the other hand, the compound with a 6-NO_2_ group showed significant activity against *Staphylococcus aureus* (MIC 12.5 μg mL^−1^) and *Escherichia coli* (MIC 25 μg mL^−1^). On the contrary, compounds bearing the benzyl group at second position of the MBT nucleus did not show any antimicrobial profile.

Analogues **47** (Figure 24), a benzothiazole-2-thiol derivative bearing amide linkages and phenyl rings, were synthesized [120] by condensing 2-chloro-*N*-arylacetamide with 6-aminobenzothiazole-2-thiol in refluxing acetone and subsequent treatment with the corresponding acyl chloride in dichloromethane in the presence of sodium bicarbonate at room temperature. Compounds were investigated for their anti-proliferative activities on human liver hepatocellular carcinoma (HepG2) and human breast adenocarcinoma (MCF-7) cells. Authors disclosed that replacement of the hydrogen at the 4-position of the phenyl ring present at the sixth position of the benzothiazole ring with an electron-attracting nitro group, resulted in complete loss of activity. However, replacing the hydrogen at the 2-position of the phenyl ring with an electron-donating methoxy group enhanced the anti-proliferative activities by approximately 57-fold against HepG2 cells, and 32-fold against MCF-7 cells.

**Fig. 25.**
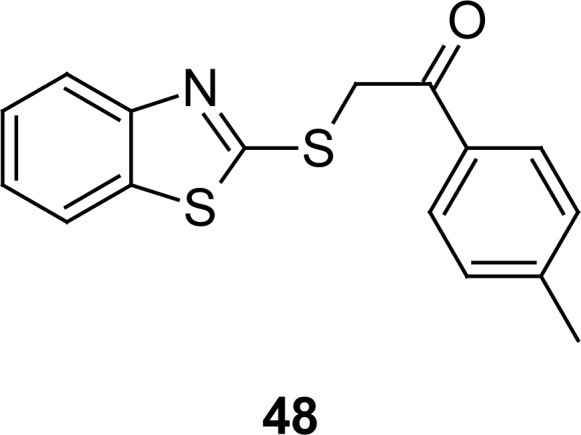
Structure of compounds that have shown chemoprotection of thymocyte apoptosis induced by dexamethasone

The chemoprotection of thymocyte apoptosis induced by dexamethasone and *γ*-irradiation is described [121] for the pifithrin-α analogue 2-(1,3-benzothiazol-2-ylsulfanyl)-1-(4-methylphenyl)ethanone (**48**Figure 25), which in turn was prepared by reaction of 2-bromo-4′-methylacetophenone with MBT, resulting exclusively in the S-alkylation product. Compound **48** showed cytoprotective activity, with an average of 16% cells remaining when challenged with dexamethasone.

**Fig. 26.**
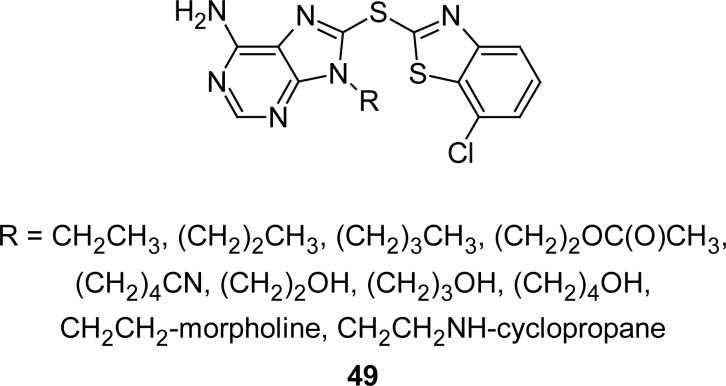
Structure of Analogues of MBT having Hsp90 inhibitory property

The discovery of benzothiazolothiopurines **49** (Figure 26) as potent heat shock protein 90 (Hsp90) inhibitors is an important development. Authors described the structure-activity relationship [122]. The benzothiazole moiety was found to be exceptionally sensitive to substitutions on the aromatic ring with a 7′-substituent essential for activity. Some of these compounds exhibited low nanomolar inhibition activity in a Her-2 degradation assay (28–150 nM), good aqueous solubility, and oral bioavailability profiles in mice. *In vivo* efficacy experiments demonstrated that compounds of this class inhibit tumor growth in an N87 human colon cancer xenograft model via oral administration as shown with compound 8-(7-chlorobenzothiazol-2-ylsulfanyl)-9-(2-cyclopropylamino-ethyl)-*9H*-purin-6-ylamine. The parent compound, which carries no substituent on the benzothiazole ring, induces Her-2 degradation in MCF-7 cells with 5000 nM IC_50_.

**Fig. 27.**
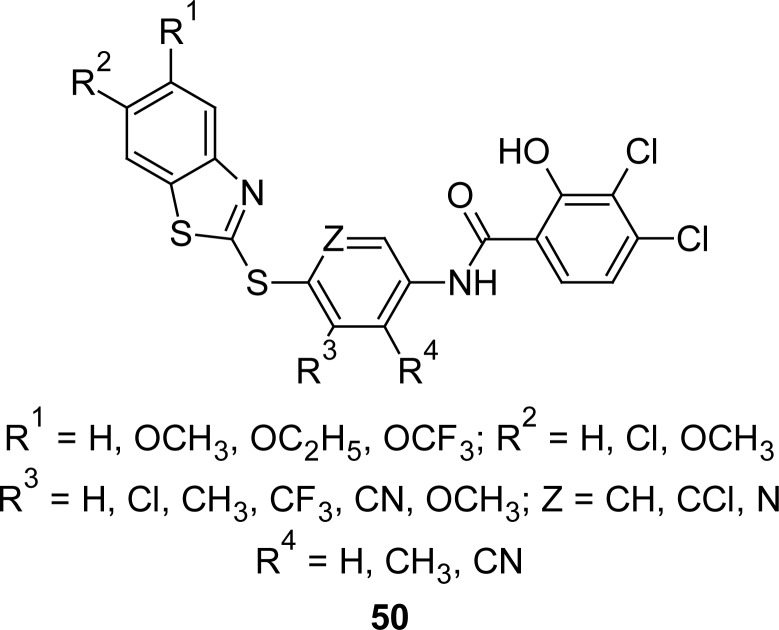
Analogues of MBT with cathepsin D inhibitory property

Cathepsin D, a lysosomal aspartyl protease, has been implicated in the pathology of Alzheimer’s disease as well as breast and ovarian cancer. Impressed by these facts, MBT analogues **50** (Figure 27) were synthesized and screened as a cathepsin D inhibitor [123]. It was observed that the heteroatom linker between the two rings can be either sulfur or oxygen, while substitution of the middle ring resulted in a slight increase in activity when a lipophilic substituent (chlorine, methyl, trifluoromethyl) is added ortho to the heteroatom linker. The overall potency of these analogues seems on track with the lipophilicity of the side-chain.

**Fig. 28.**
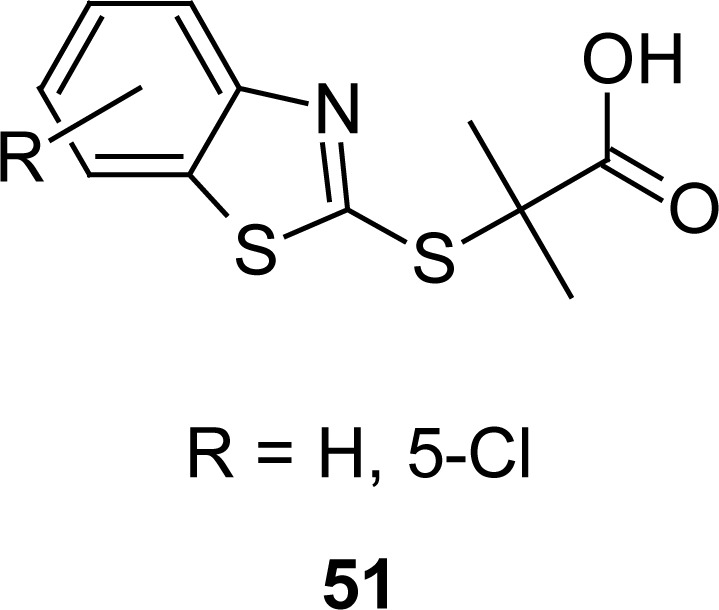
Structure of compound having promising antiplatelet activity

MBT analogues of clofibric acid **51** (Figure 28) have been synthesized by the reactions of ethyl 2-bromoisobutyrate with the sodium salts of MBT or 5-chloro-2-mercaptobenzothiazole in refluxing ethanol, and subsequently hydrolyzing the resultant esters in the presence of potassium hydroxide. Effects of compounds on platelet aggregation were evaluated using anticoagulated bull blood [124]. The 5-chloro analog showed significant anti-aggregating property, revealing a valuable dose-dependent pattern.

**Fig. 29.**
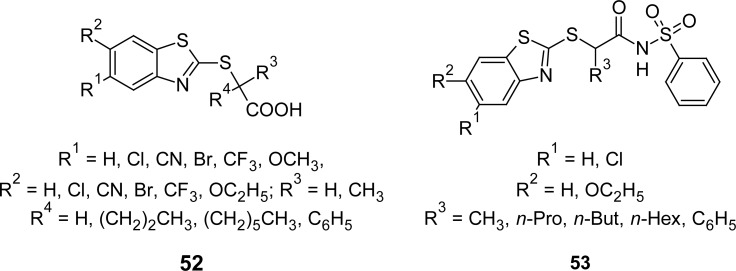
Various congeners of MBT that have shown PPARα transactivation activity

Another group of researchers synthesized a series of 2-benzothiazolylthioalkanoic acids **52** (Figure 29) through systematic structural modifications of clofibric acid and evaluated them for human PPARα transactivation activity, with the aim of obtaining new hypolipidemic compounds [125]. Overall, the potencies of some newly designed agonists were slightly higher than those of typical fibrates, such as clofibrate. While unsubstituted derivatives proved inactive, the overall effect of the introduction of substituents in the five and six positions improved PPARα agonistic activity. Among the series, the 5-bromine derivative (R^1^ = Br, R^2^ = H, R^3^ and R^4^ = CH_3_) with an EC_50_ value of 2.5 μM was found to be 10–20 times more effective than other compounds of the same series.

In continuation of the above work, *N*-(phenylsulfonyl)amides **53** (Figure 29) containing the MBT scaffold were synthesized [126] by structural modification of clofibric acid. Synthesis involves direct condensation of carboxylic acid with benzensulfonamide in the presence of 1-ethyl-3-[3-dimethylaminopropyl]carbodiimide hydrochloride and 4-dimethylaminopyridine. Phenylsulfonamides were evaluated *in vitro* against the agonistic effect of GW7647; they showed an inhibitory effect on PPARα activation, with the best compounds revealing a dose-dependent antagonistic profile. Among compounds bearing an alkyl chain in α-position to the carboxylic group, the antagonistic activity seems to improve shifting from *n*-propylic derivatives (IC_50_ 31.0 μM and IC_50_ 22.2 μM) to the n-butylic derivative (IC_50_ 19.9 μM). Further elongation of the substituent gave rise to the agonistic activity, whereas the introduction of a branched chain (i-pro) slightly decreased the antagonistic activity (IC_50_ 27.9 μM). Finally, when the alkyl chain was replaced with α-phenyl group, this resulted in increased antagonistic activity (IC_50_ 6.5 μM). The different substitution pattern of the benzothiazole system did not significantly affect the activity.

**Fig. 30.**
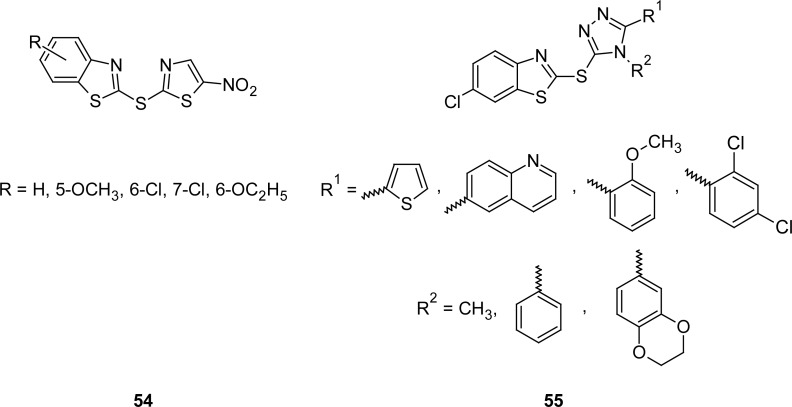
Analogues of MBT active against c-jun N-terminal kinases

The synthesis and c-Jun N-terminal kinase (JNK) inhibition activity of a novel series of 2-thioether-benzothiazoles **54** and **55** (Figure 30) is described in literature [127]. Compounds were synthesized by nucleophilic substitution of 2-bromo-5-nitrothiazole with the corresponding thiol of benzothiazole in the presence of sodium ethoxide in methanol at room temperature. The compound bearing a 2-nitrothiazole moiety at the second position of the MBT nucleus showed a promising result with an IC_50_ of 1.8 and 0.16 μmol in the Lantha screen kinase and pepJIPI DELFIA displacement assays, respectively. The activity was similar with or without methoxy, however when a chloro or ethoxy was present at the five or six position, compounds were inactive, which was likely due to steric hindrance.

## Conclusion

2-Mercaptobenzothiazoles have been widely explored for industrial applications since their discovery. However, the biological activity of this class of compounds deserves further investigation. This becomes clear when microbial infections are considered. Although the research on this subject is incipient, the number of reports disclosing the effects of MBTs on pathogens of clinical interest has recently been increasing. 2-Mercaptobenzothiazole compounds have been shown to be promising, which calls for the design of more efficient antimicrobial, anthelmintic, anti-inflammatory, and anti-allergic agents. Future studies will undoubtedly uncover unexpected properties and applications. Advances in this field will require analyses of the structure-activity relationships of MBTs, as well as the mechanisms of action of these compounds.

## Abbreviations

DELFIA: dissociation-enhanced lanthanide fluorescent immunoassay
EC_50_: half-maximum effective inhibitory concentration
GABA: gamma amnibutyric acid
GST: glutathione-S-transferase
Her-2: human epidermal growth factor receptor-2
5-HT: serotonin
5-HTR: serotonin receptor
IC_50_: median inhibitory concentration
*K*i: inhibitor constant
MIC: minimum inhibitory concentration
MAO: monoamine oxidase
MBT: 2-mercaptobenzothiazole
Nm: nanomol
PPAR: peroxisome proliferator-activated receptor.
